# A Case Report: Mitral Valve Infective Endocarditis due to *Streptococcus mitis* With Cardioembolic Stroke in the Setting of a Neuroendocrine Tumor

**DOI:** 10.1155/cric/2081665

**Published:** 2026-07-24

**Authors:** Thuy Hao Nguyen, Mahpara Munir, Ngoc Thai Kieu, Arif Karrar, Muhammad Umar, Phillip McDonald

**Affiliations:** ^1^ Internal Medicine, Hurley Medical Center/Michigan State University, Flint, Michigan, USA, hurleymc.com; ^2^ Internal Medicine, Jacobs School of Medicine and Biomedical Sciences, Buffalo, New York, USA, buffalo.edu; ^3^ Punjab Medical College, Faisalabad Medical University, Faisalabad, Punjab, Pakistan, pmc.edu.pk

## Abstract

Infective endocarditis is a life‐threatening infection of the endocardial surface, most commonly resulting from bacteremia originating from the oral cavity or intravascular sources. However, in some cases, the source of bacteremia remains unidentified, and the role of gastrointestinal pathology is not well defined. We report a patient in her 70s with multiple comorbidities, including mitral annular calcification, who presented with fever, altered mental status, and abdominal pain. Blood cultures grew *Streptococcus mitis*. Transthoracic and transesophageal echocardiography demonstrated a mobile vegetation on the mitral valve consistent with infective endocarditis. Brain magnetic resonance imaging revealed multiple acute cardioembolic infarctions. During hospitalization, persistent abdominal symptoms prompted further evaluation, which identified severe fecal impaction and a previously undiagnosed ileal neuroendocrine tumor with hepatic lesions. No recent dental procedures or other clear sources of bacteremia were identified. This case raises the possibility that gastrointestinal pathology, including bowel dysfunction and malignancy, may contribute to bacteremia and subsequent infective endocarditis. In patients without an identifiable source, targeted gastrointestinal evaluation may be considered, although further studies are needed to clarify this relationship.

## 1. Introduction

Infective endocarditis is an infection of the endocardial surface, most commonly involving cardiac valves and occurring in patients with predisposing structural abnormalities [1]. Viridans group streptococci, including *Streptococcus mitis*, are commensal organisms of the oral cavity and are a well‐recognized cause of subacute native valve endocarditis [1]. Conducting a systematic search for current and potential entry points of infectious endocarditis is crucial for effective treatment of the current episode, preventing future recurrences, and aiding in the selection of empiric antibiotics if necessary [2].

In a subset of patients, however, the source of bacteremia remains unclear. Prior reports have described associations between infective endocarditis and underlying gastrointestinal pathology, suggesting that mucosal barrier disruption may represent a potential source of bacteremia in selected cases, although this relationship remains incompletely understood [3].

We present a case of *S. mitis* mitral valve endocarditis complicated by multiple cardioembolic infarctions in the setting of severe constipation and an underlying neuroendocrine tumor, highlighting a potential but unproven gastrointestinal source of bacteremia.

## 2. Case Presentation

A patient in her 70s with a history of coronary artery disease, peripheral arterial disease, deep vein thrombosis on anticoagulation, rheumatoid arthritis on methotrexate, hypertension, hyperlipidemia, diabetes mellitus, and chronic obstructive pulmonary disease presented with abdominal pain and altered mental status. On presentation, she was febrile (38.3°C) and tachycardic (119 beats per minute).

Initial laboratory evaluation demonstrated leukocytosis. Patient met SIRS criteria for sepsis and was started empirically on broad‐spectrum antibiotics for suspected sepsis after blood cultures were obtained. Blood cultures subsequently grew *S. mitis*, and antibiotics were narrowed to ceftriaxone based on sensitivities.

**Figure 1:**
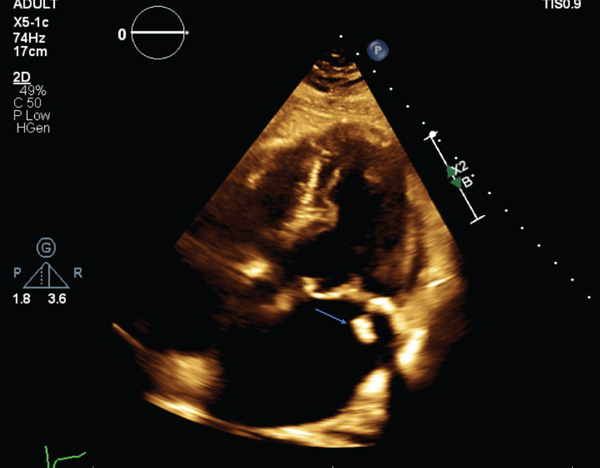
An echocardiogram showed a 1 × 1 cm mobile vegetation on the mitral valve with mild to moderate annular calcification.

**Figure 2:**
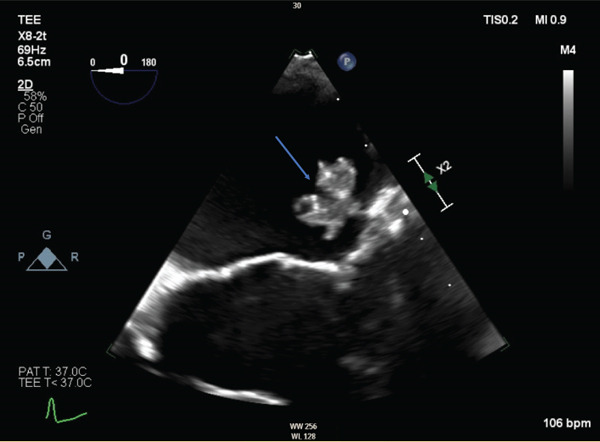
A transesophageal echocardiogram demonstrated a medium mobile mass measuring 1.43 × 0.805 cm on the mitral valve, with a dilated left atrium.

Given persistent fever and positive blood cultures, transthoracic echocardiography was performed, demonstrating a mobile vegetation on the mitral valve (Figure 1). Transesophageal echocardiography confirmed a vegetation measuring approximately 1.4 cm (Figure 2). These findings, in conjunction with positive blood cultures, were consistent with infective endocarditis.

On the first day in the hospital, the patient developed altered mentation. Brain CT was performed, showing an extra‐axial mass in the posterior fossa causing mass effect upon the left medulla, concerning for a meningioma. Brain magnetic resonance imaging was performed afterward and revealed multiple acute infarctions in both hemispheres, consistent with cardioembolic events.

Troponin elevation was noted, and coronary angiography demonstrated significant coronary artery disease. Although the patient met criteria for surgical intervention due to vegetation size and embolic complications, surgery was deferred due to comorbidities and overall clinical risk, and the patient was managed medically.

**Figure 3:**
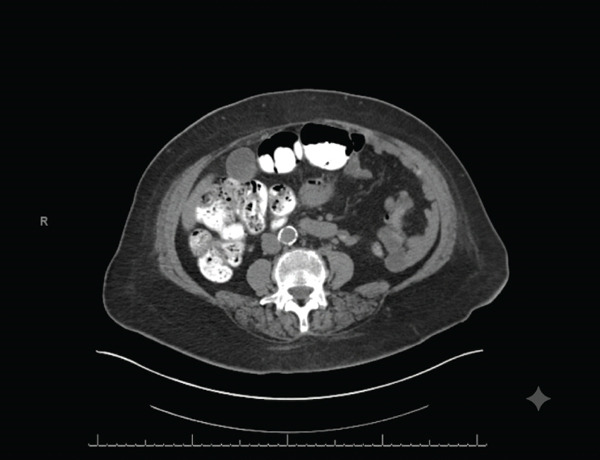
Abdominal CTA axial view confirmed the presence of a focal hypodense lesion in the distal ileal loop near the ileocecal valve measuring up to 1.9 cm.

**Figure 4:**
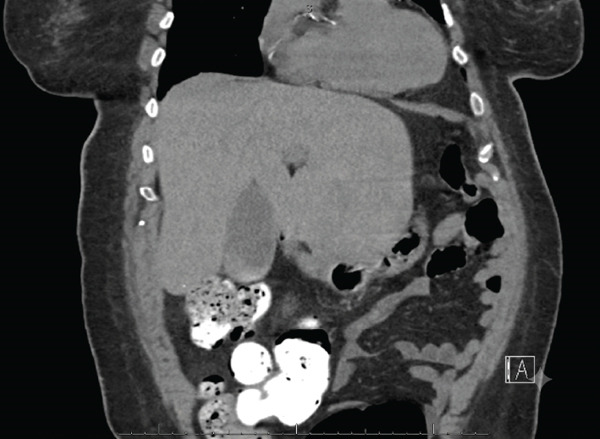
Abdominal CTA coronal view confirmed the presence of a focal hypodense lesion in the distal ileal loop near the ileocecal valve measuring up to 1.9 cm.

The patient continued to report persistent abdominal pain and constipation. Further evaluation revealed severe fecal impaction. Cross‐sectional imaging identified multiple hepatic lesions and a lesion in the distal ileum (Figures 3 and 4). Colonoscopy demonstrated a mass in the terminal ileum, and biopsy confirmed a well‐differentiated neuroendocrine tumor.

No recent dental procedures or clear oral source of bacteremia were identified. A formal dental evaluation was not documented.

The patient completed a 6‐week course of intravenous ceftriaxone 2 g daily with clinical improvement and was discharged with outpatient follow‐up for oncology evaluation.

## 3. Discussion

Infective endocarditis remains a serious condition, particularly in older patients with structural heart disease [4]. Mitral annular calcification, as present in this patient, is a recognized predisposing factor for bacterial adherence and vegetation formation [5].

Viridans group streptococci, including *S. mitis*, are commonly associated with native valve infective endocarditis and are typically linked to transient bacteremia originating from the oral cavity [1]. However, in a subset of cases, no clear source of bacteremia is identified [4]. The microbiological profile of native valve endocarditis most commonly includes *Staphylococcus aureus*, viridans group streptococci, and enterococci, whereas prosthetic valve endocarditis is more frequently associated with coagulase‐negative staphylococci and healthcare‐associated pathogens [1].

In this case, no dental or other obvious source of bacteremia was identified. The presence of severe fecal impaction and an ileal neuroendocrine tumor may have contributed to bowel stasis and mucosal barrier disruption. Intestinal stasis has been associated with bacterial translocation, potentially allowing commensal organisms to enter the bloodstream [6]. These findings raise the possibility of a gastrointestinal source of bacteremia, although a causal relationship cannot be definitively established.

Similar reports have described associations between infective endocarditis and underlying gastrointestinal pathology, particularly in the setting of mucosal disruption or malignancy [3]. However, in most instances, the relationship remains associative rather than causal, and a definitive source of bacteremia is often not identified.

The patient′s course was complicated by multiple cardioembolic infarctions, a known complication of infective endocarditis associated with large, mobile vegetations [1]. Current guidelines support surgical intervention in patients with infective endocarditis who have large vegetations (> 10 mm) and embolic complications. In this case, the patient met these criteria; however, surgery was deferred due to comorbidities and overall clinical risk.

In retrospect, this case highlights a potential missed opportunity for earlier recognition of gastrointestinal pathology. The patient′s chronic constipation and persistent abdominal symptoms may have warranted earlier evaluation. Although it is unclear whether earlier intervention would have altered the development of bacteremia or endocarditis, earlier recognition may have reduced the risk of systemic complications.

From a clinical perspective, this case does not support routine gastrointestinal evaluation in all patients with infective endocarditis. However, in patients with unexplained bacteremia or endocarditis without a clear source, particularly in the presence of persistent gastrointestinal symptoms, targeted gastrointestinal evaluation may be considered. This case primarily emphasizes diagnostic consideration rather than a change in standard management.

## 4. Conclusion

This case highlights a potential association between gastrointestinal pathology and *S. mitis* infective endocarditis. Although causality cannot be established, gastrointestinal conditions may represent a possible source of bacteremia in selected patients. Clinicians should consider a targeted evaluation when no clear source is identified, while maintaining standard diagnostic and management strategies.

## Funding

No funding was received for this manuscript.

## Conflicts of Interest

The authors declare no conflicts of interest.

## Data Availability

Data sharing is not applicable to this article as no datasets were generated or analyzed during the current study.
